# Critical Vibration and Control of the Maglev High-Speed Motor Based on *μ*–Synthesis Control

**DOI:** 10.3390/s22228692

**Published:** 2022-11-10

**Authors:** Yefa Hu, Kezhen Yang, Huachun Wu, Xinhua Guo, Nianxian Wang

**Affiliations:** 1 School of Mechanical & Electronic Engineering, Wuhan University of Technology, Wuhan 430070, China; 2 Shenzhen Research Institute, Wuhan University of Technology, Shenzhen 518057, China; 3 Hubei Provincial Engineering Technology Research Center for Magnetic Suspension, School of Mechanical & Electronic Engineering, Wuhan University of Technology, Wuhan 430070, China; 4 School of Machinery and Automation, Wuhan University of Science and Technology, Wuhan 430081, China

**Keywords:** high-speed motor, magnetic bearing, critical speed, vibration suppression

## Abstract

The Maglev motor has the characteristics of high-speed and high-power density, and is widely used in compressors, molecular pumps and other high-speed rotating machinery. With the requirements of miniaturization and high speed of rotating machinery, the rotor of the maglev motor will operate above the bending critical speed, and the critical vibration control of the flexible rotor is facing challenges. In order to solve the problem of the critical vibration suppression of the maglev high-speed motor, the system model of the maglev motor is established, the rotordynamics of the flexible rotor are analyzed and the rotor model is modal truncated to reduce the order. Then, the *μ*–controller is designed, and the weighting functions are designed to deal with the modal uncertainty. Finally, an experimental platform of the maglev motor with the flexible rotor is built to verify the effect of the *μ*–control on the suppression of the critical vibration of the maglev rotor.

## 1. Introduction

**Figure 1:**
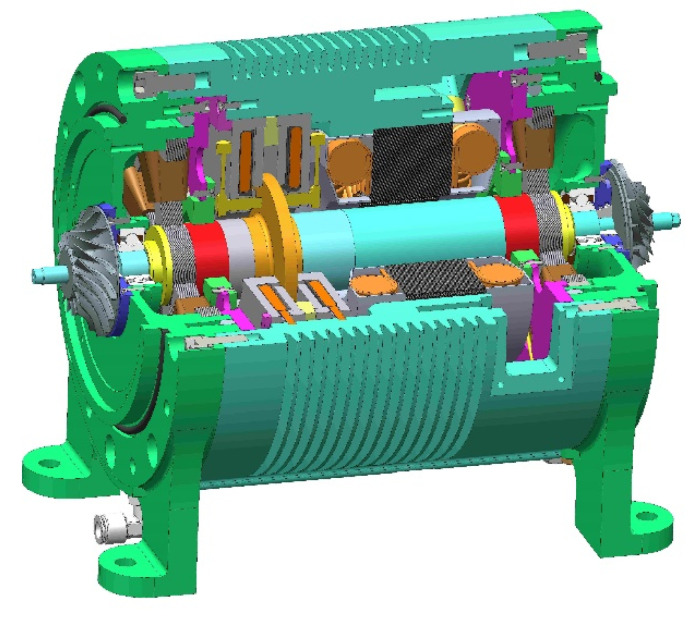
Structure of a high-speed and high-power-density maglev motor.

Active control of the magnetic field can support objects, and typical applications can be divided into two categories: maglev train [1,2,3] and active magnetic bearing (AMB) [4]. AMB has the unique advantages of high speed, no friction, and no lubrication, and has been more and more widely used in various industrial fields [5]. Especially for high-speed motors, as shown in Figure 1, AMBs eliminate friction and lubrication, reduce rotor vibration, and are more advantageous to the development of motors towards high-speed and high-power-density [6]. However, the rotor may need to operate above the bending critical frequency due to structural limitations in some applications. During the running up process of the rotor, the rotor must pass through the critical frequency, and because of the existence of unbalanced mass, the rotor system will have severe vibration, which will seriously affect the stable operation of the high-speed motor [7].

The critical vibration suppression of the flexible rotor has always been a research highlight in the field of rotating machinery such as high-speed motors. In the rotor system supported by mechanical bearings, many scholars use the squeeze film damper with elastic ring [8,9] and dynamic vibration absorber [10] to suppress the critical vibration. Ghasabi [11] applied piezoelectric fibers as sensors and actuators and implemented a nonlinear saturation-based controller with time delays to mitigate the critical vibrations of a continuous spinning shaft. Alam [12] designed a command-shaping technique for vibration control of a flexible twin rotor system.

Though for the rotor supported by the AMBs, the active controllability characteristic can be utilized with an appropriate control algorithm to suppress vibration.

Some scholars achieved control of the flexible mode of the rotor by adding a filter based on PID (proportional-integral-derivative) control. Tang [13] used a phase lead compensator and an optimal damping compensator for the 315 kW maglev motor, and in the experiment, the minimum rotor resonance response was obtained when crossing the first bending critical speed, and the maximum damping was provided when the power amplifier current was minimum. Tang [14] also proposed a mode separation method for the 100 kW maglev motor, which separated the first and second bending modes, and reconstructed the displacement near the first bending mode. The critical speed was successfully passed through with a small current in the experiment. Zheng [15] aimed at the resonance vibration problem of a 10 kW maglev centrifugal compressor with non-collocated sensors and actuators, using a notch filter and a phase lead compensator to extract the bending mode of the rotor and provide optimal damping near the modal frequency. The rotor smoothly crossed the critical speed with the low current in the experiment. Shaolin Ran [16] used a second-order phase lead filter to provide optimal damping, then designed a notch filter to eliminate the influence of a second-order bending mode, and the experiment proved its effectiveness. The design of the filters expands the application of the PID control in critical vibration control, but also increases the complexity of the control system.

The AMB-rotor system is essentially a nonlinear, strongly coupled, and multi-input-multi-output (MIMO) system. The control method based on modern control theory provides another way to suppress the critical vibration of the maglev flexible rotor. Ran [17] presented a detailed design for critical vibration suppression via mixed sensitivity H∞ control and achieved good suppression performance at the critical frequency in the experiments and prevented current saturation. Long [18] also adopted the mixed sensitivity H∞ optimization control theory and proposed a method to select the weighting function according to the amplitude at the natural frequency, which reduced the vibration at the critical speed in the experiment, and the rotor operated above the second bending critical speed. The *μ*–synthesis robust control considers the uncertainty of the model in the modeling process, and comprehensively analyzes the robust stability and robust performance of the system, which has great potential in suppressing the critical vibration of the flexible rotor [19]. Zhou [20] adopted the *μ*–synthesis control to deal with the uncertainty caused by the linearization of the magnetic force of the AMB in the molecular pump, and achieve the desired performance by designing the weighting functions. Mushi [21] studied the influence of the aerodynamic cross-coupling stiffness force on rotor instability, then designed the *μ*–controller and improved the stability of the rotor near the first bending mode.

Therefore, the current research on the critical vibration suppression of the flexible rotor of the maglev high-speed motor mainly focuses on the design of various filters, while research on modern control algorithms such as robust control is still rare. The research on the suppression of the critical vibration of the rotor of the magnetic levitated high-speed motor is very meaningful to improve the power density.

In this study, the rotor vibration control model of the maglev high-speed motor is established based on the model reduction theory, and the *μ*-controller and weighting function are designed to deal with the modal uncertainty. Finally, a maglev high-speed motor experimental platform with a flexible rotor is built to verify the effectiveness of the control algorithm in suppressing the critical vibration.

## 2. Modeling of the Maglev High-Speed Motor System

### 2.1. System Configuration

**Figure 2:**
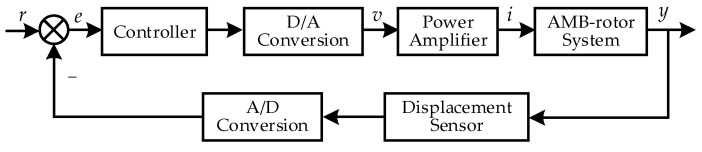
Control schematic diagram of the maglev system.

The maglev system in the high-speed motor is a closed-loop feedback system, which includes the controller, D/A conversion, power amplifier, displacement sensor, A/D conversion and AMB-rotor system. The typical control schematic diagram of the maglev system is shown in Figure 2.

**Figure 3:**
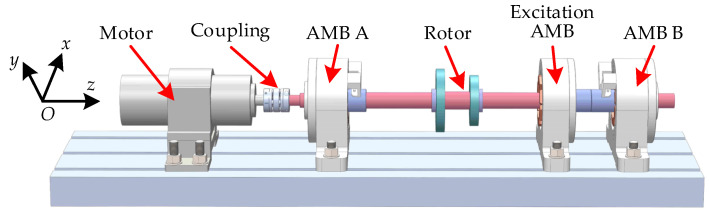
Three-dimensional model of the maglev high-speed motor.

The three-dimensional model of the high-speed motor is shown in Figure 3. The flexible rotor is supported by radial magnetic bearings at both ends, and the radial magnetic bearings in the middle are used to load external excitation to the rotor, which is not used in this experiment. The rotor is driven by a 4 kW permanent magnet synchronous motor via a lightweight flexible coupling. The maximum speed of the motor is 240 Hz. When the AMBs do not work, the rotor falls on the auxiliary bearings, which are installed beside the AMBs and can protect the stator in case of rotor instability.

### 2.2. Modeling of AMB

The AMB stator is an 8-pole heteropolar structure made of laminated silicon steel. The magnetic pole direction deviates from the vertical by 45° so that the gravity of the rotor can be evenly distributed in the two control directions, ensuring that the dynamic characteristics of the *x*-axis and *y*-axis are almost the same. In order to ensure sufficient stiffness in both positive and negative directions, the differential control mode is generally adopted. Although the electromagnetic force *F*_mag_ of the AMB is nonlinear relative to the air gap and current to simplify the control design, it can be transformed into a linear model by Taylor expansion:

(1) $$F_{\mathrm{mag}}=k_{i}i-k_{x}x$$

where, *k_i_* is the current stiffness coefficient, *k_x_* is the displacement stiffness coefficient, *i* is the control current, and *x* is the displacement of the rotor off the center.

However, due to errors in the manufacturing and assembly process, there may be differences between the actual AMB model and theoretical design model. Therefore, it is still necessary to measure the actual stiffness coefficients through a pull test [22], and in this study, the current stiffness is *k_i_* = 214 N/A and the displacement stiffness is *k_x_* = 1110 N/mm.

### 2.3. Flexible Rotor Modeling and Order Reduction

**Figure 4:**
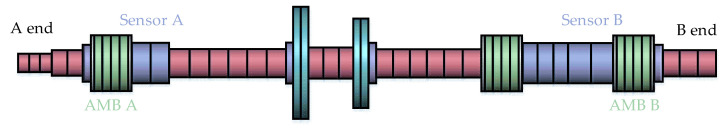
Finite element model of the flexible rotor.

When the rotor operates above the first bending mode frequency, it will become a flexible rotor. The transfer matrix method or finite element method can be generally used for the modeling of flexible rotors. Because the transfer matrix method may cause numerical instability in the calculation process, the finite element method [23,24] with higher calculation accuracy is adopted in this study. The finite element model of the flexible rotor can be obtained by dividing the rotor into 56 elements, as shown in Figure 4. The driven end of the rotor is defined as the A end, and the non-driven end is the B end.

The measuring ring, silicon steel sheet, permanent magnet, shaft sleeve and other accessory parts, which are sleeved on the outside of the spindle, can be regarded as the added mass and inertia moment on the node. Therefore, the dynamic model of the rotor can be expressed by a second-order differential equation:

(2) $$\left\{ \begin{array}{l} M\ddot{q}+(D+\Omega J)\dot{q}+Kq=Q_{1}F_{\mathrm{mag}}+Q_{2}f\\ y=Q_{3}q \end{array}\right.$$

where, ***M***, ***D*** and ***K*** are mass matrix, internal damping matrix and stiffness matrix, respectively, which are positive definite symmetric matrices. ***J*** is the gyroscopic matrix as an antisymmetric matrix. ***q*** is the displacement of each rotor element along the *x* and *y* axes and angular displacement around the *x* and *y* axes, and thus a 224 × 1 matrix. ***F***_mag_ is a column vector containing 4 elements which represent the electromagnetic force, and ***Q***_1_ is the distribution matrix of the electromagnetic force. ***f*** is the external force on the rotor, generally the unbalanced force, and ***Q***_2_ is its distribution matrix. ***y*** is the displacement output matrix of the sensor and ***Q***_3_ is the distribution matrix of the displacement sensors. For the specific acquisition method of each matrix in the finite element model, refer to the relevant rotor dynamics books [25].

**Figure 5:**
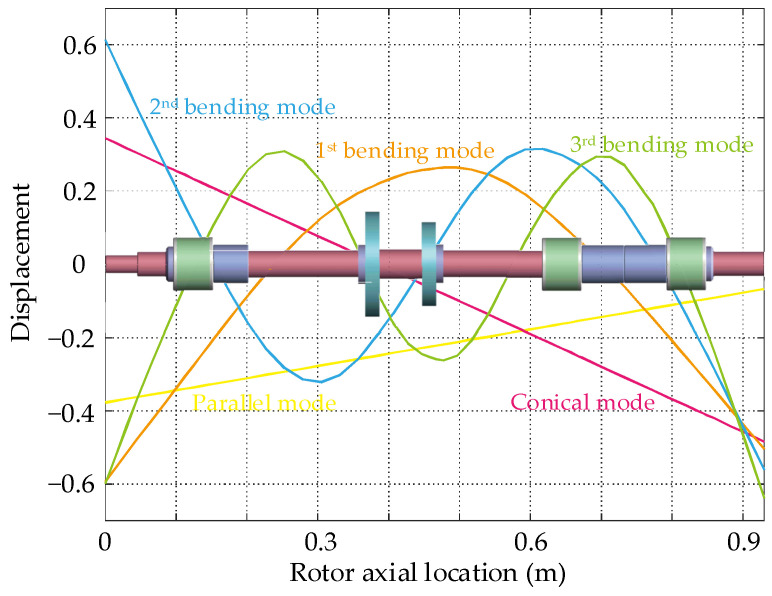
Modal shape of the flexible rotor.

According to the finite element principle, the rotor finite element model is dynamically analyzed. Rotor modal analysis is an important process in rotor design, which shows the layout of the displacement sensor position and AMB support position along the rotor in rigid body mode and bending mode. The undamped free-free modal shape of the flexible rotor is obtained by programming the dynamic characteristic calculation and demonstrated in Figure 5.

**Figure 6:**
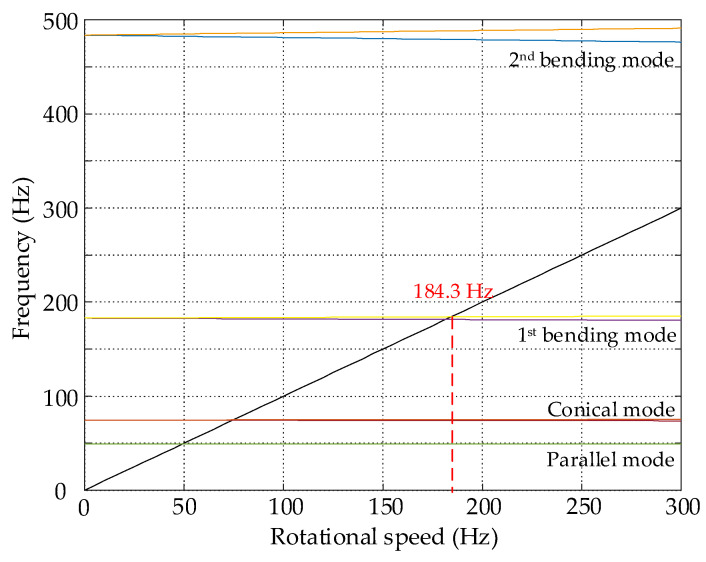
Campbell diagram of the flexible rotor.

From the point of view of control, the design of the maglev flexible rotor is reasonable. The first bending mode has good controllability and observability, the sensor-actuator non-collocation does not introduce a stability problem, which provides a prerequisite for suppressing the critical vibration at the first bending critical speed. Additionally, with the increase in the operation speed, the rotor eigenvalues will bifurcate, so the Campbell diagram is introduced to analyze the gyroscopic effect, as shown in Figure 6. The closed-loop support stiffness of the AMBs is set to 10^6^ N/m according to engineering experience. The first bending frequency of the rotor does not have obvious bifurcation with the increase in the rotational speed, indicating that the gyroscopic effect can be ignored in the controller design process. Therefore, the following modeling and analysis are only conducted in the *xOz* plane. The first critical speed of the rotor is *ω*_1_ = 184.3 Hz, and the second critical speed is *ω*_2_ = 496.1 Hz, so the rotor will pass through the first critical speed during running up.

However, the flexible rotor model in Equation (2) is developed on the physical coordinates after finite element division, and the dimension of the dynamic model is 224. It will be very difficult to design and simulate the controller based on such a model. Therefore, it is necessary to truncate the original flexible rotor dynamics model and ignore the uninterested higher-order modes to obtain the lower order model. 

To transform physical space into modal space, the modal transformation matrix ***ψ*** is constructed as

(3) q=ψη

where, the modal transformation matrix ***ψ*** is a selected subset containing feature vectors of corresponding modes, and ***η*** is the transformed modal coordinate. The modal matrix only contains a set of truncated effective modes, namely the first two bending modes in this work. Then, by substituting Equation (3) into Equation (2) and premultiplying by ***ψ***^T^ at both sides of the equation, the corresponding truncation form is obtained as follows

(4) $$\left\{ \begin{array}{l} \psi^{T}M\psi \ddot{\eta }+\psi^{T}D\psi \dot{\eta }+\psi^{T}K\psi \eta =\psi^{T}Q_{1}F_{\mathrm{mag}}+\psi^{T}Q_{2}f\\ y=Q_{3}\psi \eta \end{array}\right.$$

The reduced model in Equation (4) above can be expressed as

(5) $$\left\{ \begin{array}{l} M_{r}\ddot{\eta }+D_{r}\dot{\eta }+K_{r}\eta =\psi^{T}Q_{1}F_{\mathrm{mag}}+\psi^{T}Q_{2}f\\ y=C_{r}\eta \end{array}\right.$$

where, ***M****_r_* = ***ψ***^T^***Mψ*** = ***I***, ***D****_r_* = ***ψ***^T^***Dψ***, ***K****_r_* = ***ψ***^T^***Kψ*** = ***Λ***^2^ = diag [0, 0, *ω*_1_^2^, *ω*_2_^2^], ***C****_r_* = ***Q***_3_***ψ***. The internal damping of the metal rotor is generally very small, the damping matrix can be expressed as ***D****_r_* = 2*ξ**Λ***, where *ξ* = 0.3%. After modal truncation, modal coordinate ***η*** only contains 4 degrees of freedom, including two orders in rigid mode and two orders in flexible mode, which greatly simplifies the controller design. Then, substitute Equation (1) into Equation (5), the dynamic differential equation of Equation (5) is converted into the state space form as follows

(6) $$\left\{ \begin{array}{l} \left[\begin{array}{c} \dot{\eta }\\ \ddot{\eta } \end{array}\right]=\left[\begin{array}{cc} 0 & I\\ -\Lambda^{2}+k_{x}\psi^{T}Q_{1}Q_{1}{}^{T}\psi & -2\xi \Lambda \end{array}\right]\left[\begin{array}{c} \eta \\ \dot{\eta } \end{array}\right]+\left[\begin{array}{cc} 0 & 0\\ k_{i}\psi^{T}Q_{1} & \psi^{T}Q_{2} \end{array}\right]\left[\begin{array}{cc} i & f \end{array}\right]\\ y=\left[\begin{array}{cc} C_{r} & 0 \end{array}\right]\left[\begin{array}{c} \eta \\ \dot{\eta } \end{array}\right] \end{array}\right.$$

### 2.4. Modeling of Electrical Modules

The power amplifier is an important part of the actuator in the AMB system; it receives the signal from the controller and generates the corresponding current to drive the coil. The dynamics of the power amplifier is related to DC bus voltage and coil inductance. In order to establish its model, the method of system identification is utilized to exert the swept-frequency cosine signal at the input and measure the current value at the output to obtain its frequency response curve. The transfer function of the single channel power amplifier is obtained by curve fitting as

(7) $$G_{a}(s)=\frac{(\tau_{a}s+1)\omega_{a}{}^{2}}{s^{2}+2\xi_{a}\omega_{a}s+\omega_{a}{}^{2}}$$

where, *τ_a_* = 0.00012 s, *ω_a_* = 530 rad/s, *ξ_a_* = 0.22. The dynamic characteristics of each channel of the power amplifier are similar, which can all be expressed by the transfer function of Equation (6). Therefore, the transfer function of the two-input-two-output power amplifier in the *xOz* plane is expressed as blkdiag [*G_a_*, *G_a_*], where blkdiag represents the block diagonal matrix constructor function.

Four eddy current displacement sensors are used to measure the rotor position in real-time. The measurement range is 1 mm and the output range is 0~5 V. Since there is a low-pass filter circuit in the signal processing circuit of the sensor to eliminate high-frequency signals, the transfer function *G_s_* (*s*) of the single channel displacement sensor can also be expressed as a first-order inertial element:

(8) $$G_{s}(s)=\frac{K_{s}}{\tau_{s}s+1}$$

where, *K_s_* = 5000 V/m, *τ_s_* = 0.000056 s. Similarly, the transfer function of the two-input-two-output displacement sensor in the *xOz* plane is expressed as blkdiag [*G_s_*, *G_s_*].

## 3. *μ*–Synthesis Control Design

**Figure 7:**
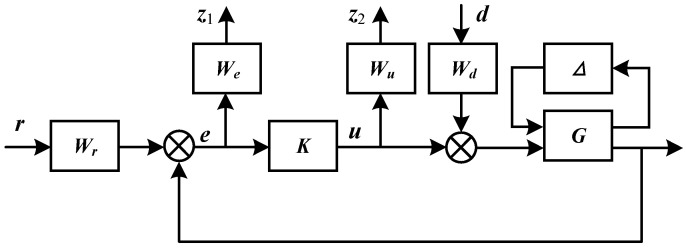
Block diagram of *μ*-synthesis control.

As a multivariable controller design technology, when considering the uncertainty of the system, *μ*–synthesis technology takes robust stability and robust performance as the unified optimization index, and is very suitable for dealing with complex MIMO control problems, such as the control of the flexible mode of the maglev rotor in this study [26]. The control block diagram of the feedback loop of *μ*–synthesis control is shown in Figure 7, where ***G*** is the physical system of the high-speed motor and ***K*** is the *μ*–controller to be designed, **Δ** is parameter uncertainty. ***r*** is the reference position signal, ***e*** is the position tracking error, ***d*** is the external disturbance, ***u*** is the controller output, ***z***_1_ and ***z***_2_ are weighted outputs, and they are all vectors. ***W_u_***, ***W_e_***, ***W_d_*** and ***W_r_*** are weighted function matrices that define the performance.

The transfer function matrix from the inputs to the performance outputs is

(9) $$\left[\begin{array}{c} z_{1}\\ z_{2} \end{array}\right]=M\left[\begin{array}{c} r\\ d \end{array}\right]=\left[\begin{array}{cc} W_{e}SW_{r} & W_{e}GSW_{d}\\ W_{u}KSW_{r} & W_{u}TW_{d} \end{array}\right]\left[\begin{array}{c} r\\ d \end{array}\right]$$

where ***S*** = ***I***/(***I*** + ***GK***) is the output sensitivity function of the closed-loop system, and ***T*** = ***GK***/(***I*** + ***GK***) is the complementary sensitivity function. *μ*-synthesis is to find a stabilizing controller ***K*** so that the structured singular value is less than 1, which is

(10) $$\mu_{\underline{\Delta }}(M):=\frac{1}{\min\left\{\bar{\sigma }(\Delta ):\Delta \in \underline{\Delta },\det(I-M\Delta )=0\right\}}<1$$

### 3.1. Modal Frequency Uncertainty

The model established by the finite element method will have errors compared with the actual rotor, and the change in interference fit characteristics of the rotor assembly during operation leads to the change in the global stiffness of the rotor, which both cause the perturbation of the modal frequency. The rotor modal frequency perturbation is a kind of parameter uncertainty, and its change will have a great impact on the robustness of the system. Proper consideration of uncertainty is the key problem of robust control. The *i*-th bending modal frequency of the nominal flexible rotor model is *ω_i_*, then the uncertain modal frequency considering the multiplicative uncertainty *δ_i_* is expressed as *ω*_i_ (1 + *δ_i_*). Since the modal frequency only appears in the state matrix ***A*** of the system state space equation in Equation (6), the uncertain state matrix with the uncertain modal frequency can be expressed as

(11) $$\begin{array}{cc} \Delta A_{i} & =\left[\begin{array}{cc} 0 & 1\\ -{\left[\omega_{i}\left(1+\delta_{i}\right)\right]}^{2} & -2\xi_{i}\omega_{i}\left(1+\delta_{i}\right) \end{array}\right]\\ & =\left[\begin{array}{cc} 0 & 1\\ -\omega_{i}{}^{2} & -2\xi \omega_{i} \end{array}\right]+\delta_{i}\left[\begin{array}{cc} 0 & 0\\ -2\omega_{i}{}^{2} & -2\xi \omega_{i} \end{array}\right] \end{array}$$

The perturbation range of the first order bending mode frequency is [−5%, +5%], and that of the second order bending mode frequency is [−2%, +2%] in this work. And since the uncertainty *δ_i_* is very small, their square terms are ignored in Equation (11).

### 3.2. Weighting Functions Design

The weighting function is generally difficult to calculate accurately or pertinently design, so it is designed based on previous experience. The forms of the weighting functions are all MIMO transfer function matrices. Since the performance requirements and dynamic characteristics of the four degrees of freedom of the flexible rotor are almost the same, the weighting function of each channel can be taken as the same. Since the gyroscopic effect of the rotor has been ignored in Section 2.3, the design is only conducted in the *xOz* plane, so the weighting function matrices are as follows

(12) $$\left\{ \begin{array}{c} W_{r}=\mathrm{blkdiag}\left[w_{r},w_{r}\right]\\ W_{d}=\mathrm{blkdiag}\left[w_{d},w_{d}\right]\\ W_{e}=\mathrm{blkdiag}\left[w_{e},w_{e}\right]\\ W_{u}=\mathrm{blkdiag}\left[w_{u},w_{u}\right] \end{array}\right.$$

where, the reference input weighting function ***W_r_*** is generally used to limit the sensitivity peak of the whole system. According to the requirements on system sensitivity in the international standard ISO 14839-3 [27], it can be selected as a constant, namely *w_r_* = 0.33 in this study. The disturbance input weighting function ***W_d_*** is to constrain the peak of the complementary sensitivity function and is generally taken as a constant from 0.2 to 1. So in this study, *w_d_* = 0.6.

The error output performance weighting function ***W_e_*** is to determine the performance of the maglev rotor such as the steady-state error, overshoot, and settling time. If designed as a constant, it can only produce an effect similar to that of a PD controller. In order to ensure steady-state accuracy, an integrator is also required, so ***w_e_*** is designed as

(13) $$w_{e}=a_{e}\frac{b_{e}s+\omega_{e}}{s+c_{e}\omega_{e}}$$

where, *b_e_* = 0.41, *ω_e_* = 30 Hz is the cross-frequency with the 0 dB line, *c_e_* = 0.0733 is the integral constant, and *a_e_* = 2.6 is the gain used to help ***W_r_*** and ***W_d_*** constrain the sensitivity function ***S*** and dynamic flexibility function ***GS***.

The control output weighting function ***W_u_*** is to limit the control sensitivity and achieve rapid attenuation of the controller gain at high frequencies to avoid control voltage saturation, so it is usually in the form of a second-order high pass filter. Generally, ***W_u_*** is the most difficult weight function to design. After several iterations, the final design is

(14) $$w_{u}=0.0102\cdot \frac{\omega_{u1}{}^{2}}{\omega_{u2}{}^{2}}\cdot \frac{s^{2}+0.707\omega_{u2}s+\omega_{u2}{}^{2}}{s^{2}+0.707\omega_{u1}s+\omega_{u1}{}^{2}}$$

where, *ω_u_*_2_ = 516 rad/s is the roll-down frequency, and *ω_u_*_1_ = 8 × *ω_u_*_2_ adds high frequency poles to ensure the reciprocal of the transfer function also exists.

### 3.3. Controller Synthesis and Order Reduction

**Figure 8:**
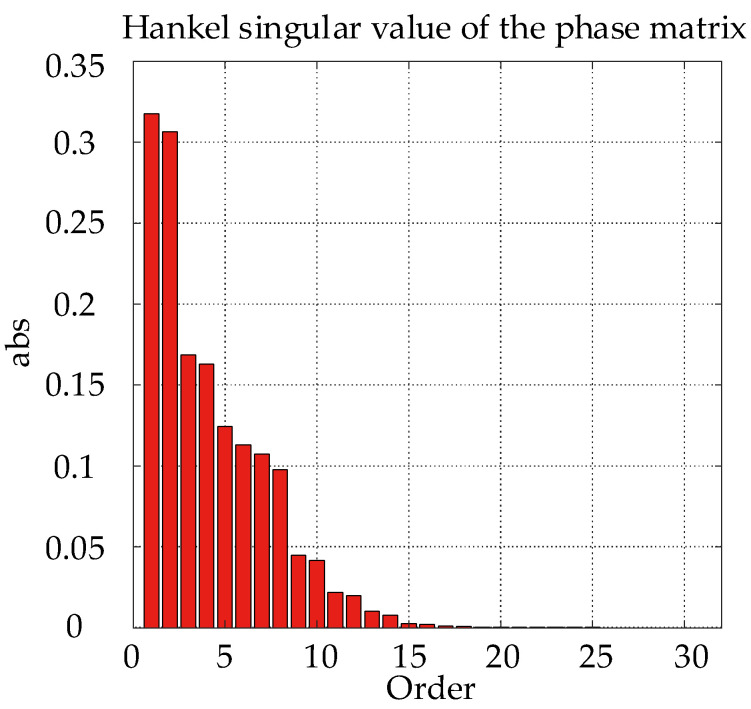
Hankel singular values of the controller.

In this study, the D-K iterative method is used for nonconvex optimization to find the optimal solution of the *μ*-synthesis problem, and the dksyn function in MATLAB is used for the iteration steps. After several iterations, a 32-order controller is obtained. Such a high order controller will bring a great burden to hardware calculation and is difficult to implement in practice. Therefore, the controller order should be reduced to eliminate the weakest controllable and observable state. The uncertainty of the system is multiplicative, and the Hankel singular values of the full order controller are shown in Figure 8.

**Figure 9:**
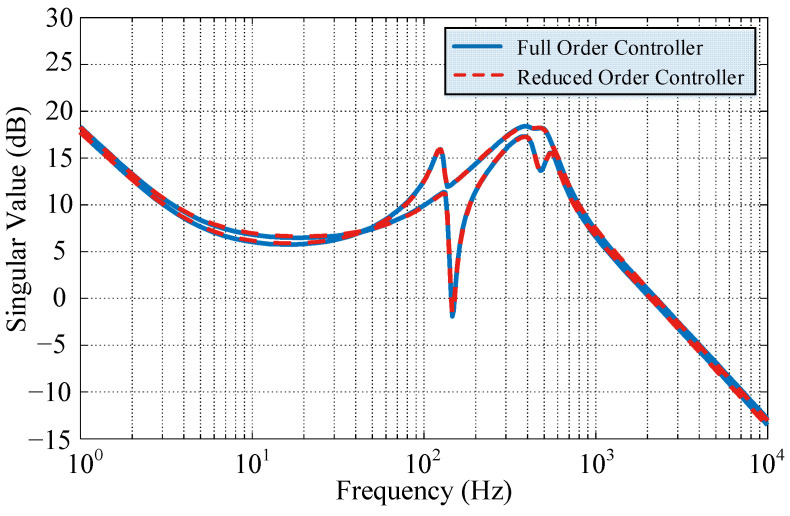
Singular values of the *μ*-controllers.

Therefore, the stochastic equilibrium order reduction method is used to reduce the controller order. According to the magnitude of the Hankel singular value in Figure 8, a 14-order controller is selected. The singular values of both the full order and reduced order *μ*-controller are shown in Figure 9, and the agreement between them is high, indicating that the order reduction does not change the robustness and control performance of the controller.

**Figure 10:**
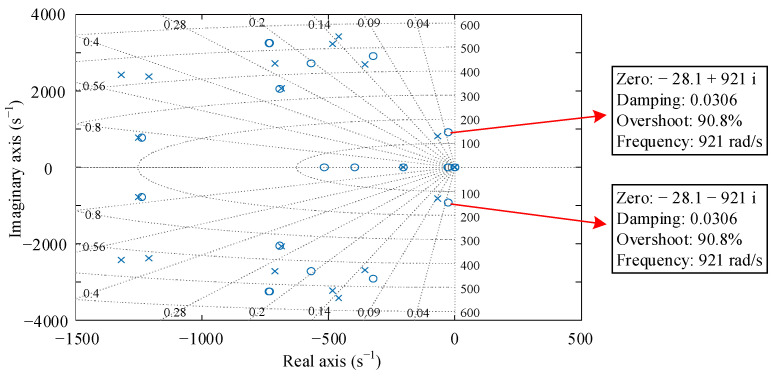
Pole-zero plot of the *μ*-controller.

The pole-zero plot of the reduced order *μ*-controller is demonstrated in Figure 10. All poles of the controller are located in the left half of the complex plane, so the *μ*-controller is stable. A pair of zeros —28.1 ± 921 i are inserted before the first bending frequency of the flexible rotor to stabilize the weak damping modal.

**Figure 11:**
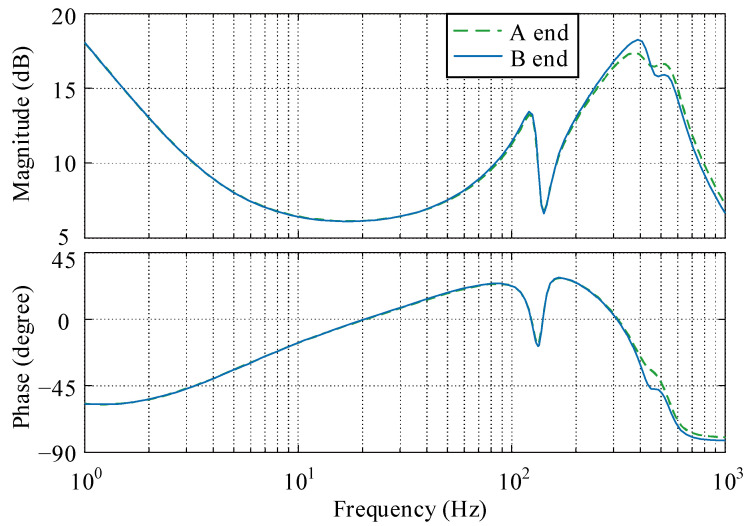
Bode diagram of the *μ*-controller.

The bode diagram of the reduced order *μ*-controller of the channels from A end to A end and from B end to B end is given in Figure 11. At low frequency, due to the limitation of the weighting function ***W_e_***, the controller has a good integration effect, which ensures high static stiffness and good steady state accuracy. In the middle frequency band, the controller is similar to the PD controller, which has a good stabilizing effect on the rigid body mode of the rotor. At high frequency, the controller has a series of poles and zeros to stabilize the flexible mode of the rotor and rapidly obtain the gain decay.

**Figure 12:**
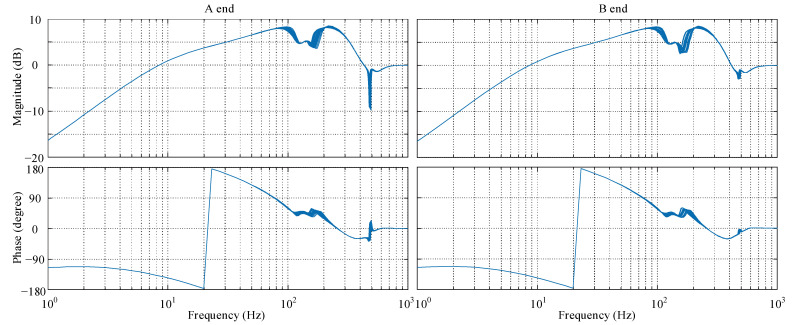
Sensitivity function of the closed-loop system.

Additionally, the sensitivity function of the closed-loop system under the *μ*-controller with uncertain modal frequency is shown in Figure 12. The sensitivity peaks of the system at the A end and B end are 8.31 and 8.25 dB, respectively. Although the uncertainty exits, the *μ*-controller still has good performance and the sensitivity function falls within Zone A according to ISO 14839-3.

## 4. Experimental Platform and Verification

**Figure 13:**
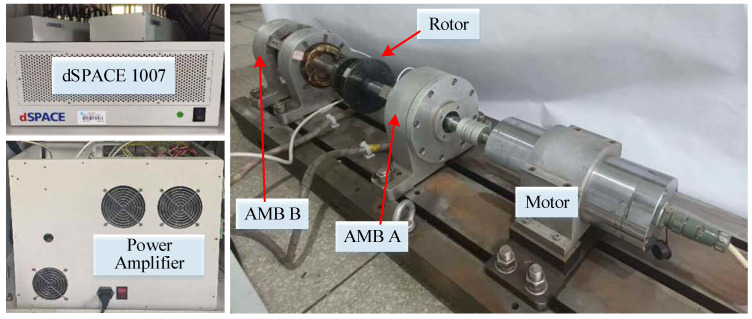
Maglev high-speed motor experimental platform with the flexible rotor.

To verify the control effect of the *μ*-controller on the critical vibration of the flexible rotor of the maglev high-speed motor, the maglev high-speed motor experimental platform with the flexible rotor is built. The experimental platform is shown in Figure 13, including the maglev high-speed motor, dSPACE rapid control prototype, power amplifier and monitoring computer.

The DC bus voltage of the power amplifier is 70 V, and the switching frequency is 25 kHz. The measuring range of the eddy current displacement sensor is 1 mm, and the static accuracy is ±0.5 μm. The parameters of the experimental platform are in Table 1. After designing the *μ*-controller according to the previous process, the controller is discretized at a frequency of 20 kHz with bilinear transformation and then executed in the dSPACE 1007 rapid control prototype.

**Figure 14:**
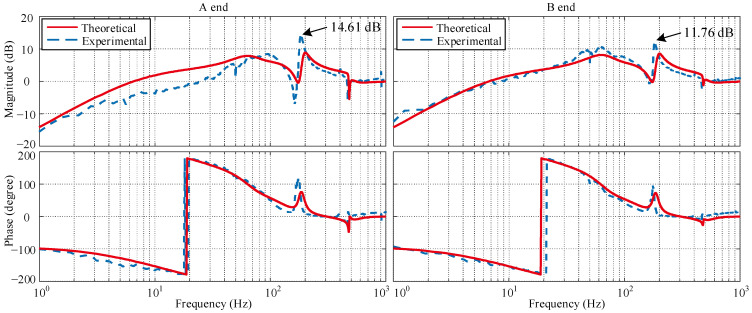
Sensitivity function comparison with the PID controller.

Before the implementation of the *μ*-controller, an ordinary PID controller with incomplete differentiation is applied as the controller in Figure 2. The system sensitivity function with PID control can be measured by the online swept-frequency experiment [27]. The swept-frequency cosine signal is in linear mode from 0.1 to 2000 Hz in 3 s, and the signal amplitude is from 50 to 80 mV. The theoretical sensitivity function can be acquired on the basis of the models in Section 2. So the sensitivity function comparison between the theoretical simulation and experimental result with the PID controller is displayed in Figure 14.

The comparison shows good agreement between the theoretical simulation and experimental results, proving the accuracy of the established models. The slight inconsistency of the A end at low frequency results from the flexible coupling, and the uncertainty of low frequency would not cause serious robustness problems. The sensitivity peaks of the A end and B end with the PID controller, occurring near the critical frequency are 14.61 and 11.76 dB, respectively, and hardly fall within the acceptable stability margin.

**Figure 15:**
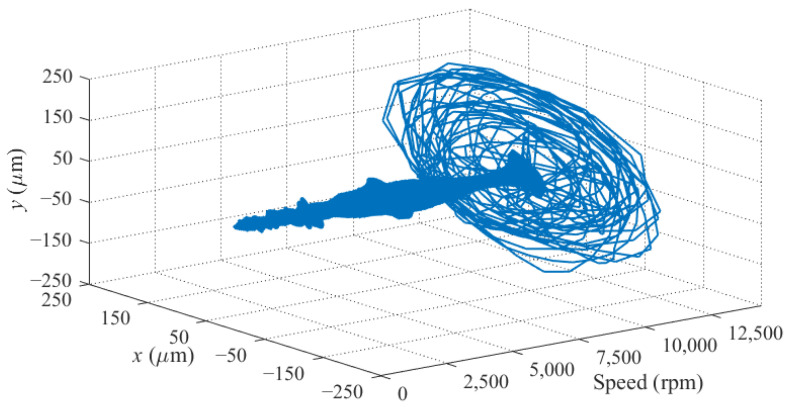
Rotor trajectory during running up with the PID controller.

The running up experiment with the PID controller is then conducted. When the rotor speed rises to near the critical speed, the rotor vibration soars drastically, and finally leads to severe contact between the rotor and auxiliary bearing at 10,960 rpm, as shown in Figure 15. The rotor fails to pass through the first bending mode frequency with the PID controller, because it can only provide little damping at the critical speed.

**Figure 16:**
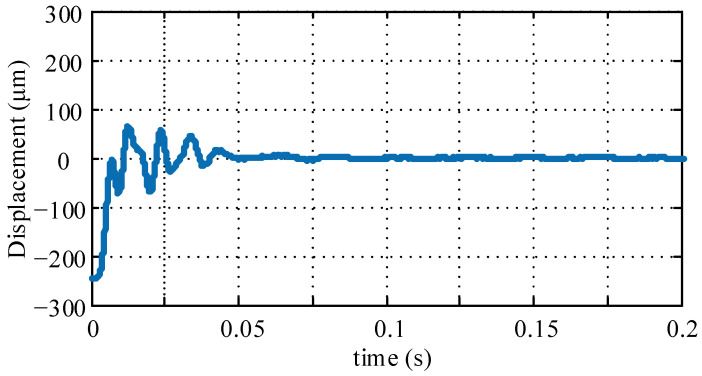
Levitation response curve with the *μ*-controller.

Furthermore, the *μ*-controller is implemented to replace the PID controller. The levitation response test of the *μ*-controller is first conducted, the rotor orbit is shown in Figure 16. The rotor levitates from the auxiliary bearing and reaches the equilibrium position in less than 0.05 s, and the overshoot is less than ±80 μm, which shows good transient performance of the controller. After stable levitation, it has high steady-state precision, within ±5 μm.

**Figure 17:**
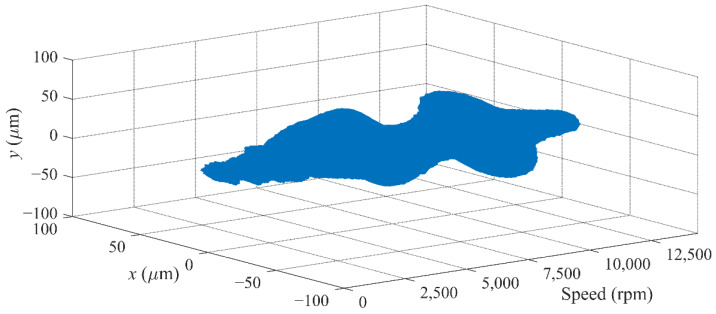
Rotor trajectory during running up with the *μ*-controller.

**Figure 18:**
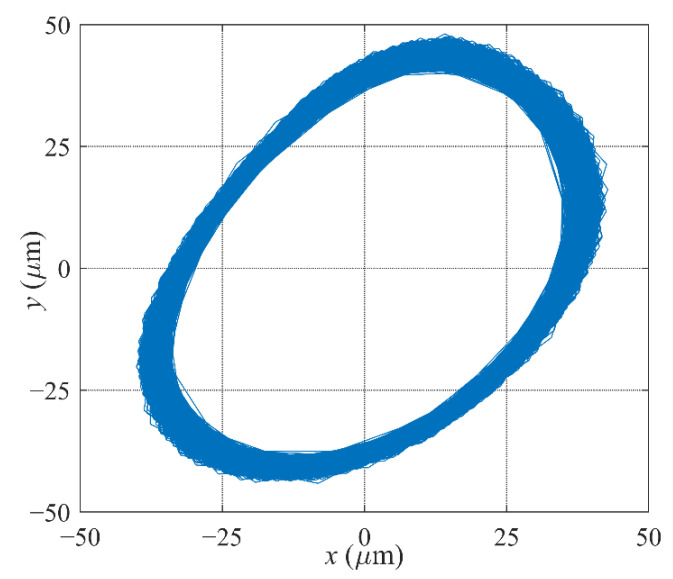
Rotor trajectory under the first critical frequency.

The running up experiment with the *μ*-controller is also conducted. Figure 17 gives the rotor orbit during the running up process. The flexible rotor passes through the first bending mode frequency of about 10,900 rpm and then stably operates at the maximum speed of 14,400 rpm. It can be clearly observed that the rotor orbit increases considerably at the rigid modal frequency of 2940 and 4500 rpm and the flexible modal frequency of 10,900 rpm. The rotor orbit at the first bending frequency, which is the largest during the running up, is shown in Figure 18. The rotor displacement is within ±50 μm, and the control effect is still good, which verifies the effectiveness of the *μ*-controller and correctness of the design process in this study.

## 5. Conclusions

This study analyzes the critical vibration suppression of the maglev high-speed motor with *μ*-synthesis control. The accurate model of the maglev system is firstly established and the dynamics of the flexible rotor analyzed. Then, the modal frequency uncertainty of the flexible rotor is mainly considered, weighting functions limiting the performance are designed, and *μ*-controller is developed and its order is reduced. Finally, the experimental platform of the maglev high-speed motor with the flexible rotor is built, and the rotor smoothly passes through the first bending mode frequency to achieve the supercritical operation.

## Figures and Tables

**Table 1:** Parameters of the experimental platform.

Parameter	Value
Rotor length	0.93 m
Rotor mass	18.14 kg
Rotor moment of inertia	0.0167 kg·m^2^
Air gap	0.5 mm
Protection gap	0.25 mm
Bias current	2.5 A
Control frequency	20 kHz
DC bus voltage	70 V
Switching frequency	25 kHz

## Data Availability

Not applicable.
